# Scale-up of CHO cell cultures: from 96-well-microtiter plates to stirred tank reactors across three orders of magnitude

**DOI:** 10.1186/s13036-024-00475-8

**Published:** 2025-01-15

**Authors:** Anne Neuss, Thomas Steimann, Jacinta Sofia Tomas Borges, Robert Dinger, Jørgen Barsett Magnus

**Affiliations:** Biochemical Engineering (AVT.BioVT), RWTH Aachen University, Aachen, Germany

**Keywords:** Scale-up, Microtiter plates, Shake flasks, Stirred tank reactor, CHO cells, OTR

## Abstract

**Background:**

For process development in mammalian cell cultivations, scale-up approaches are essential. A lot of studies concern the scale transfer between different-sized stirred tank reactors. However, process development usually starts in even smaller cultivation vessels like microtiter plates or shake flasks. A scale-up from those small shaken devices to a stirred tank reactor is barely stated in literature for mammalian cells. Thus, this study aims to address data-driven scale-up for CHO DP12 cells. The oxygen transfer rate is used as a database.

**Results:**

The cultivation conditions in microtiter plates and shake flasks are comparable when choosing the maximum oxygen transfer capacity as a scale-up parameter. The minimum cultivation volume was reduced to 400 µL in round and square 96-deep-well microtiter plates. Using a scale-up based on the maximum oxygen transfer capacity to a stirred tank reactor led to conditions with excessive hydromechanical stress. However, cultivation conditions could be reproduced in a stirred tank reactor by utilizing the volumetric power input as a scale-up parameter. Key metabolites behaved the same in all three scales and the final antibody titer was equal.

**Conclusion:**

This study presents a successful replication of cultivation results for mammalian cells in microtiter plates, shake flasks and stirred tank reactors. The working volumes ranged from 0.4 to 50 and 600 mL. It offers the opportunity to adapt the method to other, more sensitive mammalian cells and to perform cost- and time-effective experiments in high-throughput.

## Introduction

It is becoming increasingly important in the pharmaceutical industry to focus on time- and cost-saving methods during process development. Process development in large scales is elaborate. Thus, it is normally executed on lab-scale and the process is later scaled up to production scale [1]. Furthermore, it is often not feasible to explore the influences of all process parameters in large-scale experiments [2]. Therefore, scale-up and scale-down approaches are very common. The main goal of scaling up processes is to increase the working volume while keeping the product yield and quality as well as cell density and viability similar. On the other hand, scale-down models aim to mimic typical phenomena in large-scale vessels [2, 3]. Most of the scale approaches in the literature for mammalian cells focus on the scale transfers between differently sized stirred tank reactors (STR), for example in [2–7]. Studies are summarized by Lemire et al. [8]. They also describe the most important key process parameters that must be considered when scaling up. These comprise aeration, oxygen supply, CO_2_ stripping, mixing time, and hydrodynamic shear stress including power input. Ideally, all these parameters should be kept constant between different scales. However, as this is technically not possible, one parameter must be chosen as the scale-up/-down criterion. The most commonly used parameters in cell culture experiments are a constant volumetric mass transfer coefficient (k_L_a) value, a constant volumetric aeration rate, impeller tip speeds, or a constant volumetric power input (P/V) [8]. A constant k_L_a value considers oxygen supply and a constant volumetric aeration rate is based on sufficient CO_2_ stripping [8]. When using a constant P/V as scale-up parameter, gas transfer and mixing phenomena are considered. P/V is a crucial parameter for mammalian cell cultures as it is a variable to quantify hydromechanical stress. This is particularly important in STRs as the average P/V (P/V_Ø_) and the maximal local P/V (P/V_max_) occurring behind the stirrer blades have to be distinguished [9, 10]. Additionally, gas bubbles and bubble bursting must be taken into account as an influence on the cells in STRs [11].

Nowadays, miniaturized STRs are a common choice for small-scale experiments. The minimal cylindric vessels comprise 250 mL whereas vessels with squared geometry have minimal filling volumes of approximately 10 mL [12–14]. However, there are other options like shaking flasks or microtiter plates (MTP). These are very easy to handle, time- and cost-effective, and provide a high degree of parallelization capability [15]. However, these advantages can only be utilized if the results obtained are comparable to those on larger scales. For microbial systems, scale-up approaches from small shaken devices to larger STRs are a common method and reviewed by Marques et al. [16]. To our knowledge, these approaches are barely stated in the literature for mammalian cell systems. Scale transfer between MTPs, shake flasks and STRs was only found within the three publications briefly described in the following. None of them considered MTPs as small as 96-well MTPs and with different geometries. Micheletti et al. [17] performed a scale translation from shaken 24-well MTPs (800 µL) to conical flasks (100 mL) and a STR (3.5 L) with VPM8 hybridoma cells. They used a constant P/V as a scale-up parameter and found that the MTP cultivations were not completely comparable to larger scales. The growth rate was reduced while lactate concentrations were increased. The titer in MTPs was almost twice as high as in the STR and shake flasks [17]. In another study, a comparison of MTPs (2–4 mL), a miniature bioreactor (500 mL), and a STR (5 L) was performed in fed-batch mode with CHO cells. Here, a matched mixing time was used as the scale-up parameter. The results of the cultivation in all devices were very comparable as long as they were in batch mode. Slight deviations occurred in fed-batch mode [18]. Markert and Joeris [19] set up an automated MTP-based system (6–48 wells) for CHO-K1 cell cultures and compared the results to 1000 L STRs. The results shown are very comparable between the scales (viable cell density (VCD), product-, and lactate concentration). Unfortunately, however, no cultivation conditions were specified for the different scales [19]. Some other approaches focus on the comparability between shaken tubes and STRs [20–22].

For small-scale shaken systems, the low information content is often criticized. However, various systems for online monitoring of shake flasks and MTPs have been developed in recent years [15, 23]. One parameter with a high information content is the oxygen transfer rate (OTR). It can be monitored online and non-invasively in shake flasks and MTPs [24–26] and provides valuable information about cell density, cell activity, and the metabolic state of the cell cultures [27]. It was previously shown that OTR online monitoring is a suitable tool in shake flasks [28–30] and MTPs [31–33] for mammalian cells. It was also shown that hydromechanical stress by varying power inputs can be investigated in shake flasks [34]. Furthermore, it was demonstrated that growth behavior in shake flask and MTP cultivations (48 and 96 wells) are comparable when choosing the maximum oxygen transfer capacity (OTR_max_) as a scale parameter [31, 32]. Therefore, OTR monitoring is a valuable tool for mammalian cells and seems to be promising for scale-up approaches. Thus, the question arises whether it is possible to scale up mammalian cell cultivations from MTP scale to a STR cultivation using OTR online monitoring.

The aim of this study is firstly to identify the lowest working volume in 96-deep-well MTPs in which the results from shake flask cultivations can be replicated in MTP for CHO DP12 cells. Subsequently, the possibility of scale-up CHO cultivations from the small shaken systems (MTP and shake flasks) to a 1.5 L STR (600 mL working volume) was explored.

## Materials and methods

### Cell line and pre-cultures

Cells of the suspension-adapted CHO DP12 cell line (clone#1934, ATCC CRL-12445) were stored in the vapor phase of liquid nitrogen. For pre-cultures, one vial was rapidly thawed (for the specific method see [32]) and transferred to the cultivation medium TCX6D (Sartorius, Goettingen, Germany). This is a chemically defined medium that was supplemented with 8 mM of glutamine (Sigma Aldrich/Merck, Darmstadt, Germany). For pre-cultures, additionally, 200 nM of methotrexate (MTX, Sigma Aldrich/Merck) was added to the culture medium. This should prevent the loss of the transgene for the anti-IL-8 antibody production. All main-culture experiments did not contain MTX.

Pre-culture experiments were all conducted in a Kuhner incubator (ISF1-X Kühner AG, Birsfelden, Switzerland) shaken at 140 rpm with a shaking diameter of 50 mm. The temperature was set to 36.5 °C, humidity to 70%, and the CO_2_ concentration to 5%. The cultivations were performed in non-baffled 250 mL polycarbonate shake flasks (Corning, Glendale, USA) with vent caps. The filling volume was between 20 and 50 mL. Every 3rd to 4th day, the pre-culture was diluted to a cell density of approximately 3 × 10^5^cells mL^−1^.

### Main-cultures

All main-culture experiments were started from the shake flask pre-cultures (see Cell line and pre-cultures). The seeding cell density was set to 5 × 10^5^ cells mL^−1^. All experiments were performed at 36.5 °C.

#### MTP cultivations

The MTP cultivations were all conducted in the µTOM (micro(μ)-scale Transfer-rate Online Measurement) device [25] which was mounted in a Kuhner ISF1-X incubator (Kühner AG). Shaking conditions were set to 850 rpm at 3 mm shaking diameter. The µTOM device was flushed with gas from a gas cylinder (5% CO_2_ in synthetic air). To prevent evaporation, the gas was humidified through a washing bottle. For cultivations, 96-deep-well MTPs were used and sealed with a sterile cover (AreaSeal film, Excel Scientific, USA). In this study, round-well plates (Round-Deep well plate, 96 U-bottom well, rimless, height 42.4 mm; VWR, Darmstadt, Germany) and square-well plates (Riplate® SW 96, PP, 2 mL; Ritter, Schwabmünchen, Germany) were used. Both MTPs were equipped with U-bottoms. The filling volume was varied from 200 µL to 1000 µL. If offline analyses (see Sample preparation and offline analysis) were performed, 3 wells per condition were sampled from the MTP in the µTOM device. The MTP was removed from the incubation hood for no more than 15 min. Sampling was simultaneously with the shake flask sampling. The MTP was then placed back into the device and cultivated further.

#### Shake flask cultivations

The shake flask cultivations were conducted in a Kuhner ISF1-X incubator (Kühner AG). Shaking conditions were 140 rpm at 50 mm shaking diameter for all cultivations with one exception. For the shake flask experiment with increased P/V_Ø_ (see Fig. 4), the shaking frequency was set to 350 rpm at 50 mm shaking diameter. Humidity was set to 70% and CO_2_ concentration to 5% in the incubator. All shake flasks were non-baffled 250 mL Kuhner TOM glass flasks (Kühner Shaker GmbH, Herzogenrath, Germany). The filling volume was 50 mL. For each experiment, six shake flasks were connected to the Kuhner TOM system (Kühner Shaker GmbH). Three of these were used solely for online monitoring. The other three were sampled daily (1.5 mL) for offline analysis.

#### STR cultivations

The STR cultivations were performed in a 1.5 L Applikon ez2-Control reactor (Getinge, Gothenburg, Sweden). The filling volume was 600 mL. The aeration rate was set to 0.2 vvm using a ring sparger and a gas mixture of 5% CO_2_ in synthetic air (ca. 19.95% O_2_ and 74.1% N_2_). The reactor was equipped with a single six-blade Rushton turbine. The stirrer speed was set to 360 rpm for the first cultivation (see Scale-up from small shaken vessels to a STR based on OTR_max_) and between 100 and 250 rpm for the second one (see Scale-up of CHO cell cultivations to a STR with constant P/V_Ø_). The calculation of the stirrer speed is described in the chapter Calculations. The dissolved oxygen tension (DOT) was measured with an Applikon LumiSens sensor (Getinge). Antifoam SE15 (Sigma Aldrich/Merck) was diluted at 1:10 and added on demand. Samples were taken daily for offline analyses.

### OTR determination

The OTR was monitored in all main-culture experiments in MTPs and shake flasks. For shake flask cultivations, the Kuhner TOM (Transfer-rate Online Measurement) device (Kühner Shaker GmbH) was used which is a technology based on the RAMOS (Respiration activity monitoring system) system [24, 35]. The OTR monitoring in the 96-deep-well plates was conducted in the µTOM device [25]. The measuring principles of both devices are similar and have been previously described in detail [24, 25, 36]. For both devices, the entire measurement cycle was set to 60 min. The measuring phase was set to 18 min for the TOM device and to 20 min for the µTOM device according to [32]. The flow of gas for every shake flask in the TOM device was 11 mL min^−1^ and the total flow in the measurement compartment of the µTOM device was set to 52.5 S mL min^−1^. Because the OTR monitoring is temperature-dependent, outliers occur after opening the incubator hood. These outliers (one measuring point) were removed from the data. Please refer to Fig. S1 for exemplary original data with outliers. For the STR cultivations, the off-gas analysis BlueVary (BlueSense, Herten, Germany) was utilized to measure the oxygen and carbon dioxide concentrations which were then used to calculate the OTR.

### Sample preparation and offline analysis

#### Sample preparation

The culture broth of the main-cultures was used directly after sampling for determination of VCD and viability. The leftover culture broth was centrifuged at 2000 g for 3 min (mini centrifuge Rotilabo, Carl Roth, Karlsruhe, Germany). For further analysis, the supernatant was stored at −20 °C.

#### VCD and viability

VCD and viability were determined by two different techniques, namely the Neubauer Chamber method and the CEDEX device. Samples were stained with erythrosin B for the Neubauer Chamber method. VCD and viability were calculated from manually counted cells in four quadrants of a Neubauer Chamber (C-Chip, Neubauer improved, Carl Roth). For the automated cell counting, 300 µL of the culture broth was filled into cups. The cups were then placed into a CEDEX AS20 device (Roche, Basel, Switzerland). VCD and viability were determined by the trypan blue exclusion method.

#### Glucose and lactate concentrations

The HPLC system Dionex Ultimate 3,000 (Thermo Scientific, Waltham, USA) was used for the determination of glucose and lactate concentrations in the supernatant. A refractive index detector (RefractoMax 520, Shodex, Munich, Germany) was utilized for detection. Separation was realized by an organic acid resin column (Rezex ROAOrganic Acid H + (8%), 300 × 7.8 mm, Phenomenex Inc., Torrance, USA). The flow rate was set to 0.8 mL min^−1^ and the temperature to 40 °C. As mobile phase, a 5 mM H_2_SO_4_ was used. Separation was done in isocratic mode.

#### Glutamine concentration

The L-Glutamine / Ammonia (*Rapid*) kit (Megazyme Ltd., Bray, Ireland) was used according to the manufacturer’s instructions to determine glutamine concentrations in the supernatant.

#### Antibody concentration

The IgG antibody concentration was quantified with an in-house protocol and a Chromolith® Protein A column (4.6 × 25 mm, Sigma Aldrich/Merck) with a pore size of 300 Å.

## Calculations

For shake flasks, OTR_max_ was determined by the empirical correlation in Eq. 1 [37].

1 $${\text{OTR}}_{\text{max},\ \text{SF}}=3.27 \times {10}^{-7}\times {\text{Osmol}}^{0.05}\times {\text{n}}^{(1.18-\frac{Osmol}{10.1})}\times {\text{V}}_{\text{L}}^{-0.74}\times {\text{d}}_{0}^{0.33}\times {\text{d}}^{1.88}\times {\text{p}}_{\text{R}}\times {\text{y}}_{\text{O}2}^{*}$$

Equation 1 contains the following variables: osmolality (Osmol) [Osmol kg^−1^], shaking frequency (n) [rpm], filling volume (V_L_) [mL], shaking diameter (d_0_) [cm], maximum flask diameter (d) [mm], reactor pressure (p_R_) [bar], and oxygen mole fraction in the gas phase (y*_O2_) [mol mol^−1^]. OTR_max_ in round 96-deep-well MTPs was calculated according to Dinger et al. [25].

2 $${\text{OTR}}_{\text{max}, 96-\text{well}-\text{MTP}}=0.008\times {\text{V}}_{\text{L}}^{-1.00}\times {\text{d}}_{0}^{0.40}\times {\text{n}}^{1.00}$$

Equation 2 comprises the filling volume (V_L_) [mL], the shaking diameter (d_0_) [mm], and the shaking frequency (n) [rpm].

The OTR for STRs can be calculated by a balance around the gas bubbles (Eq. 3) and by a balance around the whole reactor (Eq. 4) using the following variables: volumetric mass transfer coefficient (k_L_a) [h^−1^], oxygen solubility (L_O2_) [moL L^−1^ bar^−1^], reactor pressure (p_R_) [bar], oxygen mole fraction at the reactor outlet (y_O2,out_) [mol mol^−1^], oxygen mole fraction equivalent to the dissolved oxygen in the liquid (y_L_) [mol mol^−1^], molar gas volume at standard conditions (V_m_) [L mol^−1^], volumetric gas flow rate at standard conditions at reactor inlet (q_in_) [vvm], oxygen mole fraction in the gas supply (y_O2,in_) [mol mol^−1^], volumetric gas flow rate at standard conditions at reactor outlet (q_out_) [vvm]. Using yO_2,out_ in Eq. 3 is correct when perfect mixing in the bioreactor is assumed. This is a valid assumption for small bioreactors.

3 $${{\text{OTR}}_{\text{STR}}=\text{k}}_{\text{L}}\text{a}\,{\times\,\text{L}}_{\text{O}2}\times {\text{p}}_{\text{R}}\times {(\text{y}}_{\text{O}2,\text{out}}-{\text{y}}_{\text{L}})$$

4 $${\text{OTR}}_{\text{STR}}=\frac{1}{{\text{V}}_{\text{m}}}({\text{q}}_{\text{in}}\times {\text{y}}_{{\text{O}}_{2},\text{in}}-{\text{q}}_{\text{out}}\times {\text{y}}_{{\text{O}}_{2},\text{out}})$$

With Eqs. 3 and 4 and the assumption that y_L_ = 0 and RQ (respiratory quotient) ~ 1 and therefore q = q_in_ = q_out_, OTR_max_ can be calculated for STRs according to Eq. 5.

5 $${\text{OTR}}_{\text{max},\text{ STR}}=\frac{{\text{k}}_{\text{L}}\text{a }\,{\times \text{ L}}_{\text{O}2 }\times {\text{ p}}_{\text{R}} \times {\text{ q }\times \text{ y}}_{\text{O}2,\text{in}}}{{\textrm{k}}_{\textrm{L}}\textrm{a } \times {\textrm{ L}}_{\textrm{O}2 }\times {\textrm{ p}}_{\textrm{R}} \times {\textrm{ V}}_{\textrm{m}}+\textrm{q}}$$

The k_L_a value for STRs can be calculated according to [37] by Eq. 6 with the constants C = 0.32, *α* = 0.74 and *β* = 0.42. The superficial gas velocity u_g_ [m s^−1^] is calculated by using Eq. 7 (pressure (p) = 1 bar, reactor diameter (d_R_) [m]).

6 $${\mathrm k}_{\mathrm L}\mathrm a=\;\mathrm C\;\times\;\left(\frac{\mathrm P}{\mathrm V}\right)^{\mathrm\alpha}\;\times\;\mathrm u_{\mathrm g}^{\mathrm\beta}$$

7 $${\text{u}}_{g}=\frac{\text{q}\times {\text{V}}_{\text{L}}\times \text{p}}{\textrm{A}\times {\textrm{p}}_{\textrm{R}}}\, \textrm{with}\, \textrm{A}=\frac{\uppi }{4 }\,{{\times\,\textrm{d}}_{\textrm{R}}}^{2}$$

The ungassed Power number (Po_ungassed_) is almost equal to the gassed Power number (Po_gassed_) for low superficial gas velocity [38] and can be calculated by Eq. 8 with the liquid density (*ρ*) [kg m^−3^], the stirrer speed (n) [s^−1^], the stirrer diameter (d) [m^−1^] and the filling volume (V_L_) [m^3^].

8 $${\textrm{Po}}_{\text{ungassed}}=\frac{{\textrm{P}}_{\text{gassed}}}{\uprho\;{ \times \textrm{ n}}^{3}\times {\textrm{d}}^{5}}=\frac{{\left(\frac{\textrm{P}}{\text{V}}\right)}_{\text{gassed}}\times {\textrm{V}}_{\text{L}}}{\uprho\;{\times \;\textrm{n}}^{3}\times {\textrm{d}}^{5}}$$

With Eq. 9, the Reynolds number (Re) can be calculated (dynamic viscosity of a fluid (ɳ) [Pa s]).

9 $$Re=\;\frac{p\;\times\;n\;\times\;\mathrm d^2}{\eta}$$

Calculation of the minimum shaking frequency for MTPs that leads to full mixing was performed according to Duetz et al. [39, 40]. Firstly, the filling height (h) was calculated according to Eq. 10 with the filling volume (V_L_) [L] and the vessel diameter (d) [mm]. For square 96-deep-well MTPs d = 8 mm.

10 $$h= \frac{{V}_{L}}{{(d)}^{2}}$$

The force ratio was then calculated by using the filling height (h) [mm] and the vessel diameter (d) [mm] (Eq. 11).

11 $$\frac{{centrifugal\;force}}{{gravitational\;force}}= \frac{2 h}{\text{d}}$$

Finally, the minimum shaking frequency, which will provide enough force for full mixing, can be calculated by Eq. 12 with the shaking diameter (d_0_) [mm], the vessel diameter (d) [mm] and the diameter factor (y). For square 96-deep-well MTPs d = 8 mm and y = 2.

12 $$n= \sqrt{\frac{\frac{{centrifugal\;force}}{{gravitational\;force}}}{5.6 \times {10}^{-7} \times \left({ d}_{0}+\frac{d}{y}\right)}}$$

## Results and discussion

### Cultivation of CHO DP12 cells in shake flasks and round 96-deep-well MTPs

**Fig. 1:**
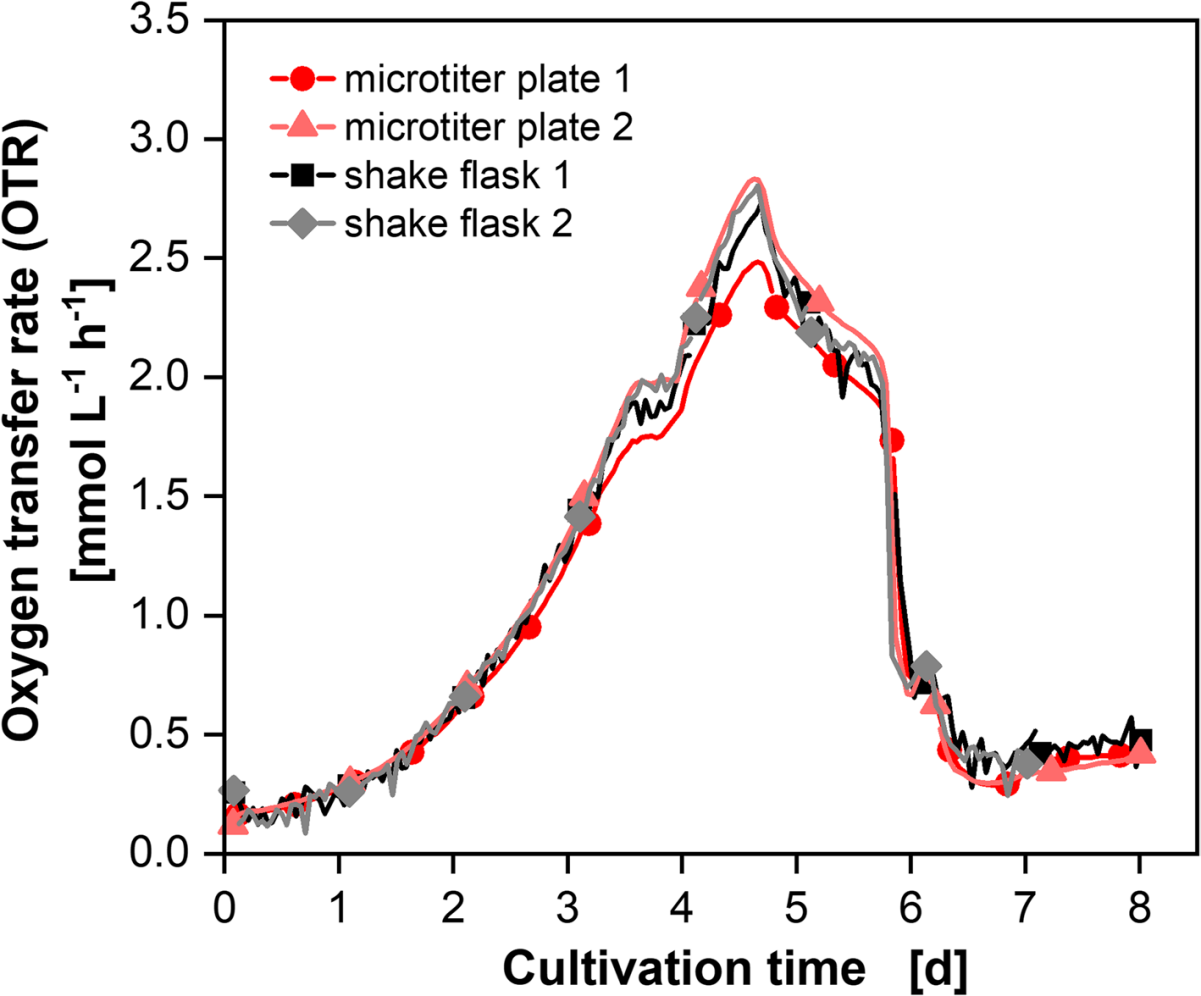
Oxygen transfer rate (OTR) of CHO DP12 cell cultures. Two independent experiments (1 and 2) were performed. For both experiments, a round 96-deep-well microtiter plate and 250 mL shake flasks were inoculated. The µTOM device was used for online monitoring of the microtiter plates (dark red line and circles; light red line and triangles) and the TOM device for the shake flasks (black line and squares; grey line and diamonds). For clarity, only every 24th measuring point over time is marked as a symbol. The microtiter plate experiments were performed in 72 (Experiment 1) and 66 (Experiment 2) replicates and the shake flask experiments in 3 replicates each. For clarity, the low standard deviations are not shown in this figure but can be found in Fig. S2. Culture conditions TOM device: 250 mL glass flasks, temperature (T) = 36.5 °C, shaking frequency (n) = 140 rpm, shaking diameter (d_0_) = 50 mm, filling volume (V_L_) = 50 mL, 5% CO_2_, 70% rel. hum., medium: TCX6D + 8 mM glutamine; starting cell density: 5 × 10^5^ cells mL^−1^. Culture conditions µTOM device: round 96-deep-well microtiter plate, temperature (T) = 36.5 °C, shaking frequency (n) = 850 rpm, shaking diameter (d_0_) = 3 mm, filling volume (V_L_) = 1 mL, 5% CO_2_, humidified, medium: TCX6D + 8 mM glutamine; starting cell density: 5 × 10^5^ cells mL^−1^

In order to verify the scale-up approach between MTPs and shake flasks for CHO DP12 cell cultures, two independent experiments were conducted. In both experiments, a round 96-deep-well MTP and shake flasks were inoculated with CHO DP12 cells and the OTR was monitored online. A scale transfer between MTPs and shake flasks was already shown in our previous publication for CHO-K1 cells with the OTR_max_ as a scale-up parameter. The cultivation conditions were chosen so that an OTR_max_ of 10.2 mmol L^−1^ h^−1^ for shake flasks and 10.5 mmol L^−1^ h^−1^ for MTPs was calculated [32]. The resulting shaking conditions (140 rpm at 50 mm shaking diameter with a filling volume of 50 mL for shake flasks and 850 rpm at 3 mm shaking diameter with 1 mL filling volume for MTPs) were adopted for the experiments shown here in Fig. 1.

As can be seen from Fig. 1, the OTR curves of all four cultivations match very well. Results are reproducible between different experiments over different scales. The low standard deviations of the OTR curves for the single experiments are shown in Fig. S2 indicating good reproducibility. The OTR curves show a first increasing phase for around 3 cultivation days until a kink is seen and the second increasing phase starts reaching a maximum of about 2.7 mmol L^−1^ h^−1^. This kink indicates glutamine depletion as was described in [32, 34]. After a decreasing phase of OTR between days 4 and 6, a sharp drop (after 5.7 cultivation days) is seen marking the time of glucose depletion as previously described [32, 34]. The cells experience equal conditions in all cultivations indicated by the similarity of the measured OTR progressions in shake flasks and MTPs. The OTR is directly correlated to the VCD [29]. The VCDs of the data presented here are illustrated in Figs. 3 and 4 and show no statistically significant difference (ANOVA, *p*-value < 0.05). It could thus be shown that an OTR_max_ based scale-up is also suitable for CHO DP12 cells. In the following, it will be investigated whether similar results are reachable with working volumes smaller than 1 mL but also on a larger scale.

### Defining minimal filling volumes for CHO cell cultivations in 96-deep-well MTPs

**Fig. 2:**
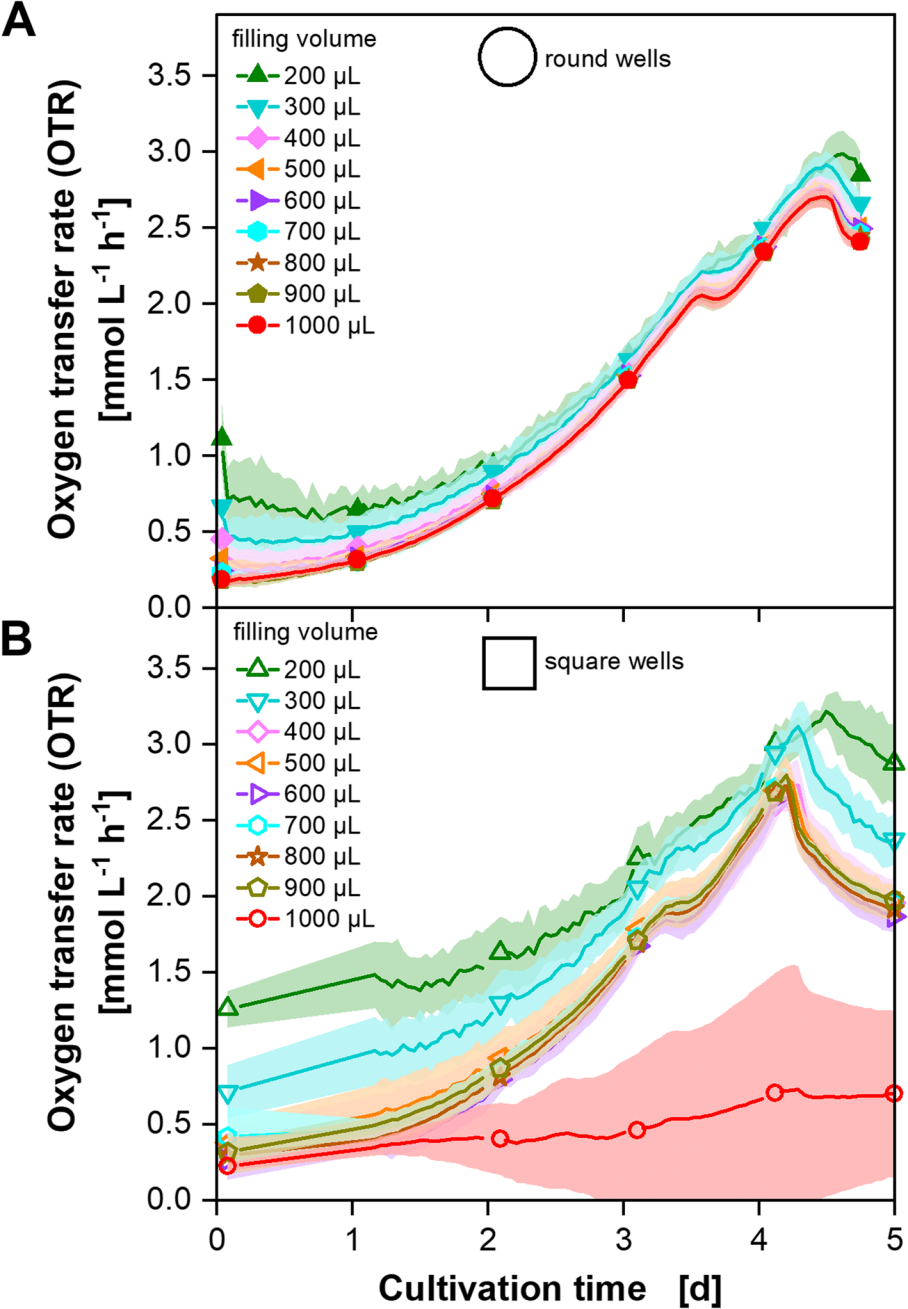
Oxygen transfer rate (OTR) of CHO DP12 cell cultures monitored by the µTOM device. **A** OTR curves of cultivations in a round 96-deep-well plate with different filling volumes (*N* = 8 for each filling volume). **B** OTR curves of cultivations in a square 96-deep-well plate with different filling volumes (N = 3 or 6 for each filling volume). For clarity, only every 24th measuring point over time is marked as a symbol. The standard deviations are shown as shaded areas. Culture conditions: temperature (T) = 36.5 °C, shaking frequency (n) = 850 rpm, shaking diameter (d_0_) = 3 mm, varying filling volume (V_L_), 5% CO_2_, humidified, medium: TCX6D + 8 mM glutamine; starting cell density: 5 × 10^5^ cells mL−1

The µTOM device was used to online monitor CHO DP12 cell cultures in 96-deep-well plates with round and square geometry with different filling volumes. The results are shown in Fig. 2.

Figure 2A shows that the OTR curves of the cultivations with 400 to 1000 µL filling volume in round well-plates are essentially equal. The standard deviation of the OTR measurement increases with decreasing filling volume. This effect was described by Dinger et al. 2022. Regarding the two main engineering parameters – OTR_max_ and P/V –, the equal results can be explained as follows: The OTR_max_ increases with decreasing filling volume (Table S1). This means that oxygen limitation does not occur in any case. The decisive factor when regarding the OTR_max_ is that no oxygen limitation occurs. It is not necessary to achieve exactly the same OTR_max_ values with different cultivations. At the same time, P/V increases with lower filling volumes [41, 42]. For 96-well MTPs, this was shown by Montes-Serrano et al. [42]. However, they also showed that P/V is not the sole criterium influencing the conditions in the fluid. In the study of Montes-Serrano et al., shear rates were simulated by computational fluid dynamics (CFD). They increase with increasing well size whereas P/V decreases with increasing well size resulting in conditions with the same P/V but shear rates up to 1000 times different. Therefore, no direct inference from P/V on culture conditions can be made. Similar results were found by Peter et al. [43] for shake flasks. They showed that the filling volume has no impact on hydromechanical stress whereas it was previously shown that P/V and the filling volume behave anti-proportionally [41]. For the data shown here, it is therefore also explainable why the same OTR curves result from different filling volumes. The cultivations with 200 and 300 µL slightly differ from the other ones—especially at the beginning of the cultivation, when the OTR values are lowest. In these two conditions, the gas volume is large compared to the oxygen consumed by the cells. This leads to an imprecise measurement of the partial pressure decrease in the wells. Therefore, these filling volumes are not recommended without adapting the measurement time. To increase the measurement precision for lower filling volumes, longer measurement phases are necessary [25].

Regarding Fig. 2B, in which the same experiment with square-well-plates is shown, similar results are seen. The OTR curves of the cultivations with filling volumes of 400 to 900 µL are again essentially equal. The ones with 200 and 300 µL are again not comparable to the other results for the reasons mentioned above. Moreover, 1 mL filling volume is not suitable for square-well plates. It leads to unpredictable and unreproducible results as can be seen by the large standard deviations indicated by the red shade. When shaking MTPs it is important to ensure full mixing so that the cells remain in suspension. It must be generated enough force to ensure that the surface of the liquid contacts the bottom of the well. This can be analyzed by using the liquid angle (see Eq. 10–12). The calculated values are depicted in Table S2 for each filling volume. The calculations show that complete mixing should no longer be possible starting from 800 µL upwards for square 96-deep-well MTPs under the cultivation conditions used in this study. However, the calculations are based on wells with a flat bottom. Round bottoms were used in this experiment. In addition, phenomena such as frictional forces are not considered in calculations and geometry of the wells leads to baffling effects which are not predictable. It can therefore be assumed that the force in wells with 1 mL filling volume is no longer sufficient to achieve complete mixing, which leads to unreproducible results. When comparing all cultivations in round and square well plates with filling volumes between 400 and 900 µL (see Fig. S3), it becomes obvious that the use of round and square geometries leads to very similar results. The OTR increases in both cases for about 4 days ending in the maximal reached OTR of about 2.7 mmol L^−1^ h^−1^. Only the shape of the peak is different (Fig. S3). The reason for the different shapes cannot be explained in detail up to now but may be due to different power inputs. Square-well plates are known for higher stress on the cells as the corners function as baffles [44]. The baffling effect is higher for square well MTPs with flat bottom compared to the MTPs used in this study with U-bottom. However, as these experiments show, they barely influence the OTRs of the tested CHO DP12 cells. The comparable OTR progressions of the different cultivations are a good hint for same behavior of the cells. However, the cellular response of the CHO cells to the conditions in the different MTPs (e.g. gene expression) cannot be seen from OTR and need further investigation.

### Scale-up from small shaken vessels to a STR based on OTR_max_

**Fig. 3:**
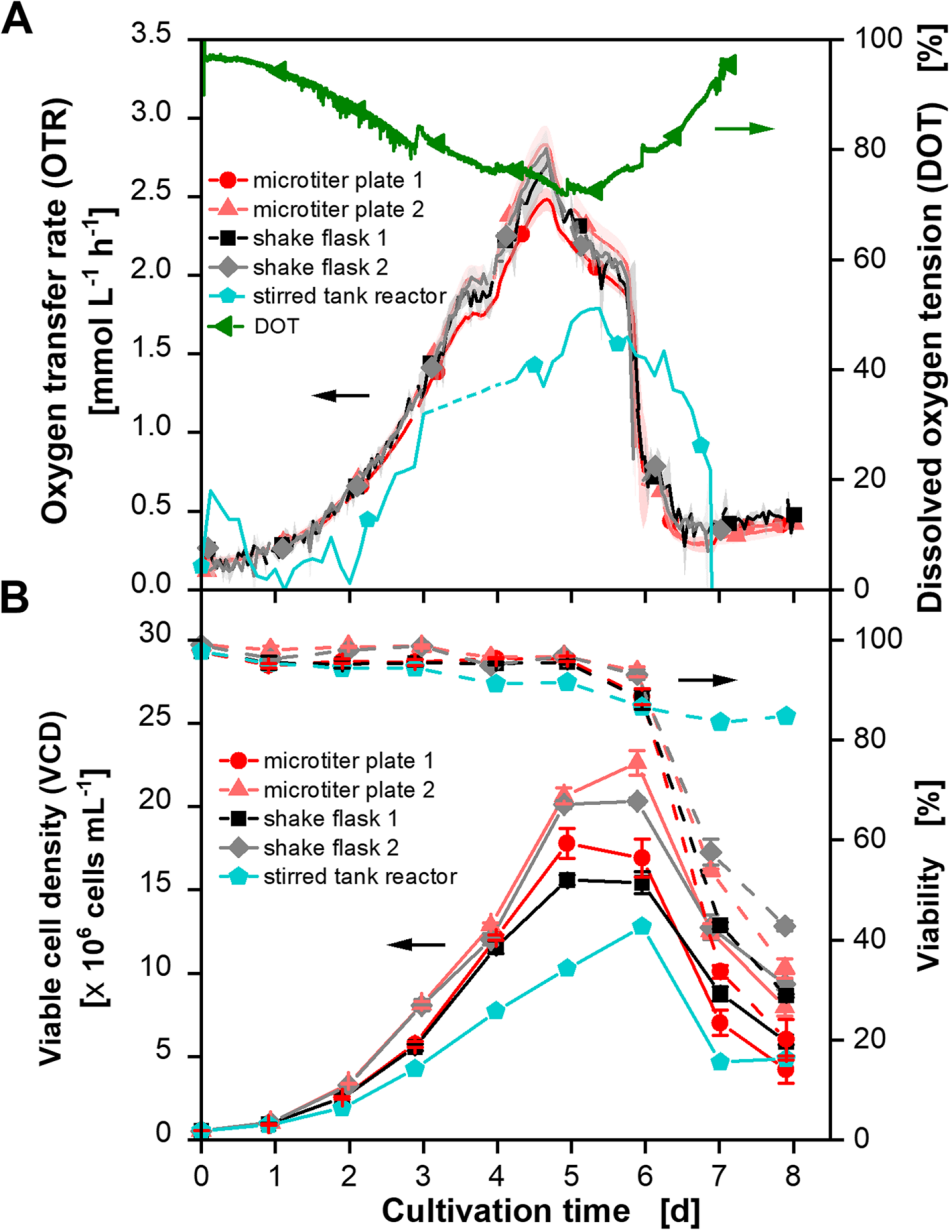
CHO DP12 cell cultivations in round 96-deep-well plates (dark and light red lines/circles and upward triangle), shake flasks (black and gray lines/squares and diamonds), and a stirred tank reactor (STR, blue line and pentagon). **A** Depicted is the oxygen transfer rate (OTR). The curves of the microtiter plate and shake flask cultivations are already shown in Fig. 1 and plotted here again for improved comparability. The data for the STR are interpolated over 3 h. The calculated OTR values between days 3 and 4 are distorted by a short-term failure of aeration and stirring and were therefore excluded from the data. For original data refer to Fig. S5 A. The dissolved oxygen tension (DOT) (green line and sideward triangle) of the stirred tank reactor is also plotted. For clarity, only one measuring point per day is plotted. **B** Displayed are the viable cell densities (VCD) and viabilities for all cultivations. Culture conditions TOM device: 250 mL glass flasks, temperature (T) = 36.5 °C, shaking frequency (n) = 140 rpm, shaking diameter (d_0_) = 50 mm, filling volume (V_L_) = 50 mL, 5% CO_2_, 70% rel. hum., medium: TCX6D + 8 mM glutamine; starting cell density: 5 × 10^5^ cells mL^−1^. Culture conditions µTOM device: round 96-deep-well microtiter plate, temperature (T) = 36.5 °C, shaking frequency (n) = 850 rpm, shaking diameter (d_0_) = 3 mm, filling volume (V_L_) = 1 mL, 5 % CO_2_, humidified, medium: TCX6D + 8 mM glutamine; starting cell density: 5 × 10^5^ cells mL^−1^. Culture conditions stirred tank reactor: 1.5 L reactor, temperature (T) = 36.5 °C, stirrer speed (n) = 360 rpm (Rushton turbine), filling volume (V_L_) = 600 mL, 5% CO_2_, aeration = 0.2 vvm (sparged), medium: TCX6D + 8 mM glutamine; starting cell density: 5 × 10^5^ cells mL^−1^

After the comparability between MTPs and shake flasks was shown, the question of whether a scale-up to a STR is also possible is addressed. The aim here is to reproduce the results shown in Fig. 1 in a STR. Therefore, a scale-up parameter is needed. In the first approach, OTR_max_ was used as a scale-up parameter as this was already successfully utilized for the scale transfer between MTP and shake flasks. For calculations, OTR_max_ for the STR was fixed to 3.75 mmol L^−1^ h^−1^ because the maximal OTR measured in the shake flask was about 3 mmol L^−1^ h^−1^ (see Fig. 1) and the DOT should not fall below 20% [8]. With this assumption, the k_L_a value was calculated according to Eq. 5 and then used to calculate P/V with Eq. 6. The resulting P/V is 0.33 kW m^−3^. The Po for one Rushton turbine is 5.4 in a turbulent flow regime (Re > 10^4^) [45]. Using this and the calculated target P/V, the stirrer speed for the experiment can be calculated by using Eq. 8. All values needed for calculation are depicted in Table S3. The resulting stirrer speed is 359 rpm. Therefore, a stirring speed of 360 rpm was used for the first scale-up experiment depicted in Fig. 3. The assumption of a turbulent flow regime was checked by determining Re. As this is > 10^4^ (1.2 × 10^4^) for the described stirring conditions, the assumption of a turbulent flow regime was valid.

Cell culture media and cultivations foam strongly in agitated systems. It was therefore not possible to avoid antifoam agents in the STR. Antifoams consist of solid hydrophobic particles, an oil, or a mixture of those [46]. It is known that antifoams can incorporate into the cell membranes and alter the permeability. This can lead to cell death or at least reduction in cell growth [47]. Flynn et al. (2024), for example, found that Antifoam 204 is toxic for CHO cells. In contrast, antifoam C led to a reduction in growth, and Antifoam SE-15 showed no inhibition [46]. Therefore, it is of high importance to wisely use antifoam in cultures. As antifoams are not additionally added to the shaken systems, the influence of antifoam SE15 on the OTR of the cultivation was tested in advance. This was carried out in MTPs with the µTOM device using different amounts of antifoam (dilutions of 1:50 to 1:10000). The results are shown in Fig. S4. The antifoam does not affect cell growth. The OTR curves with dilutions up to 1:1000 show nearly identical progressions. In a dilution of 1:500, the OTR curve is slightly shifted downwards from cultivation days 3 to 5. In a dilution of 1:100, the increase of the OTR has slowed and the maximal reached OTR is about 0.3 mmol L^−1^ h^−1^ lower than for the other cultures. In the lowest tested dilution (1:50) the OTR does not increase. It stays almost on a constant level over the whole cultivation time indicating that the cells do not grow through toxic effects of the antifoam. In the STR cultivation, the dilution was not lower than 1:1000 meaning that the influence of the antifoam on the OTR should be negligible. The results of the STR cultivation are shown in Fig. 3. The OTR curves of Fig. 1 are plotted again for better comparability.

OTR determination in shaken systems works according to the RAMOS principle, i.e. the OTR is calculated from the slope of the oxygen partial pressure decrease in the measuring phase of the system and is therefore independent of absolute oxygen concentration values. Additionally, the measurement precision can be tuned by prolonging the measurement phase [35]. This makes the OTR determination more precise compared to the off-gas analysis which uses absolute oxygen concentration values. Looking at Fig. 3, this difference in accuracy becomes obvious when comparing the OTR curves of the already described cultivations in MTPs and shake flasks (see Cultivation of CHO DP12 cells in shake flasks and round 96-deep-well MTPs) with the one of the STR (blue line and pentagons). However, it is apparent that the shape of the curves is similar but the increase of the STR-OTR curve is slower than the other ones. Additionally, the maximum reached OTR for the STR is only about 1.8 mmol L^−1^ h^−1^ compared to about 2.8 mmol L^−1^ h^−1^ for the shaken devices. The calculated OTR values between days 3 and 4 are distorted by a short-term failure (< 10 min) of aeration and stirring and were therefore excluded from the data. Oxygen availability was not influenced as the DOT did not drop below 70% (green line and sideward triangle). For calculations of the stirrer speed with the scale-up parameter OTR_max_, the requirement was made that DOT should not drop below 20%. Thus, the k_L_a value was underestimated with the correlation used here as it does not drop below 70%. The underestimation is probably because the calculation has been established for large-scale STRs where surface to volume aeration is lower than in smaller STRs and surface aeration may play a role [37].

The VCD and viability in Fig. 3B show the same trend as the OTR curves. The increase of the VCD is slower for the STR than for the shaken devices. At the same time, viability stays above 80% for at least 8 cultivation days compared to 6 for the shaken devices. This is probably because less cells were grown in the STR in overall compared to the shaken devices. Therefore, nutrients (other than glucose and lactate) are still available in the medium. Under these conditions, cells stay alive but are not able to grow anymore. Also, the peak VCD with about 12 × 10^6^ cells mL^−1^ is the lowest for STR cultivation. A slowed depletion of nutrients was also observed. Glucose was depleted one day later for the STR than for the shaken devices. The lactate switch occurred later as well (see Fig. S6 B and C). Finally, the antibody concentration did not reach the expected maximum of 250 mg L^−1^ (see Fig. S6 D). All these observations were recently published and discussed for shake flask cultivations with increased P/V and respective energy dissipation rates. Glucose and glutamine depletion, the lactate switch and the specific growth rate correlated linearly with logarithmically plotted average energy dissipation rate [34]. Therefore, it was concluded that the cultivation conditions chosen here with the scale-up parameter OTR_max_ led to conditions with too high power input. The cells were subjected to hydromechanical stress leading to a change in nutrient consumption and slowed growth. Thus, OTR_max_ based scale-up is not suitable when scaling up cell cultures from shaken devices to a STR but the hydromechanical stress in the form of P/V must be considered.

### Scale-up of CHO cell cultivations to a STR with constant P/V_Ø_

**Fig. 4:**
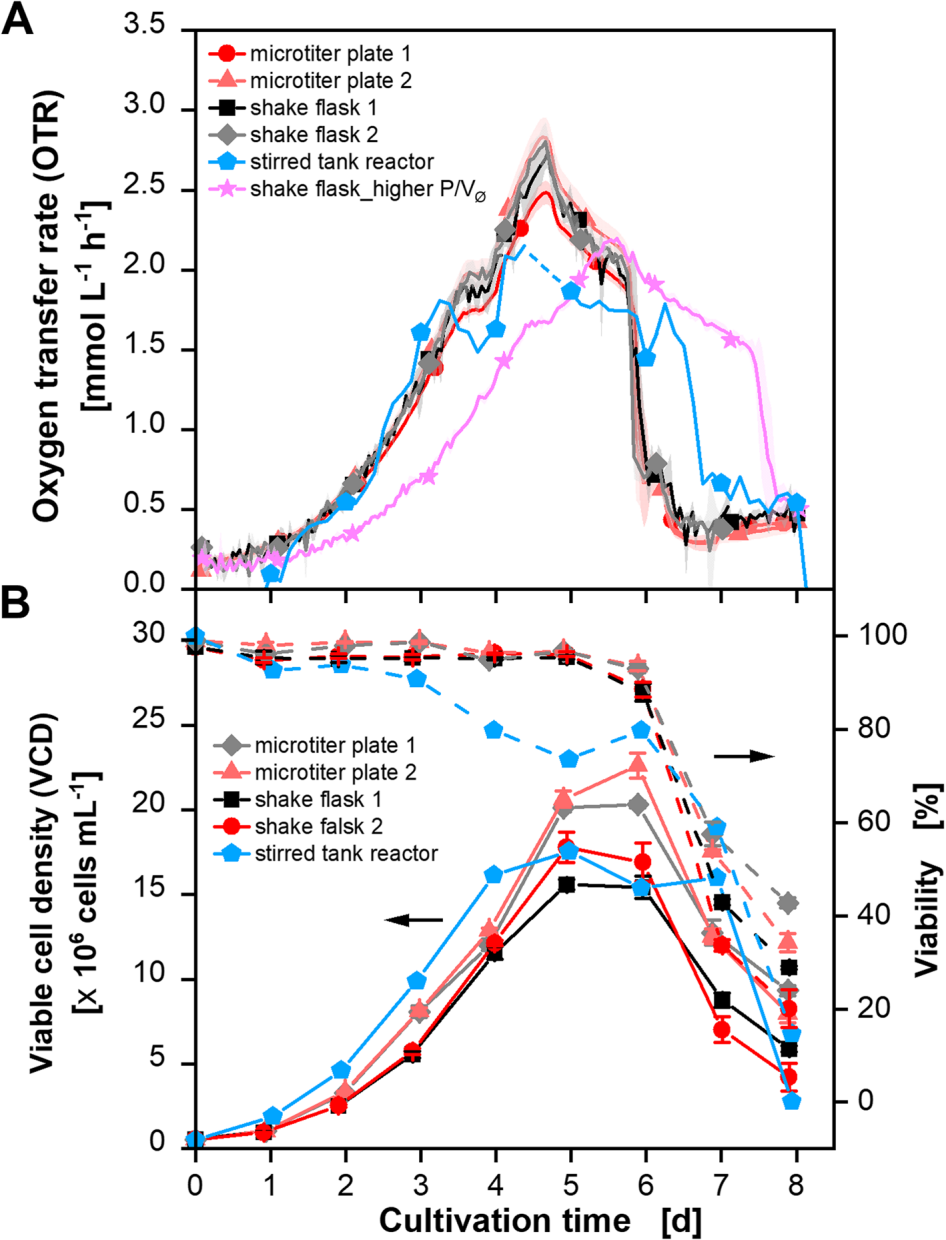
CHO DP12 cell cultivations in round 96-deep-well plates (dark and light red lines/circles and upward triangle), shake flasks (black and gray lines/squares and diamonds), and a stirred tank reactor (blue line and pentagon). **A** Depicted is the oxygen transfer rate (OTR). The curves of the microtiter plate and shake flask cultivations are already shown in Fig. 1 and plotted here again for improved comparability. Additionally, the curve of a shake flask cultivation with increased volumetric power input (P/V) from Neuss et al. [34] is shown. The data for the STR are interpolated over 3 h. For original data refer to Fig. S5 B. For clarity, only one measuring point per day is shown. **B** Displayed are the viable cell density (VCD) and viability for all cultivations. Culture conditions TOM device: 250 mL glass flasks, temperature (T) = 36.5 °C, shaking frequency (n) = 140 rpm, shaking diameter (d_0_) = 50 mm, filling volume (V_L_) = 50 mL, 5% CO_2_, 70% rel. hum., medium: TCX6D + 8 mM glutamine; starting cell density: 5 × 10^5^ cells mL^−1^. Culture conditions µTOM device: round 96-deep-well microtiter plate, temperature (T) = 36.5 °C, shaking frequency (n) = 850 rpm, shaking diameter (d_0_) = 3 mm, filling volume (V_L_) = 1 mL, 5% CO_2_, humidified, medium: TCX6D + 8 mM glutamine; starting cell density: 5 × 10^5^ cells mL^−1^. Culture conditions stirred tank reactor: 1.5 L reactor, temperature (T) = 36.5 °C, stirrer speed (n) = 100–250 rpm (Rushton turbine), filling volume (V_L_) = 600 mL, 5% CO_2_, aeration = 0.2 vvm (sparged), medium: TCX6D + 8 mM glutamine; starting cell density: 5 × 10^5^ cells mL^−1^

Due to the findings in the previous chapter, P/V_Ø_ was used as a scale-up parameter for the second experiment. The aim was to match the P/V_Ø_ of 0.12 kW m^−3^ in STR that prevails in the shake flasks under the shaking conditions in this study [34]. Therefore, Eq. 8 was used to calculate the stirring speed with a P/V_Ø_ = 0.12 kW m^−3^ and the parameters used before. The resulting stirrer speed is about 250 rpm. In STRs, the height of P/V is dependent on locality. There are regions with higher local P/V behind the stirrer blades and regions with lower P/V. Each cell experiences different power inputs which are combined in the average P/V (P/V_Ø_). To not expose the cells to a too high P/V at the beginning of cultivation, the assumption was made to not exceed the maximal local P/V. In literature, different estimations of the deviation of P/V_max_ from P/V_Ø_ are made. Kresta and Wood [48] for example showed that P/V_max_ is about tenfold higher than P/V_Ø_. Therefore, a second criterium was set for the beginning phase (as long as DOT was above 80%) of the cultivation. The upper limit of P/V (P/V_max_) should not exceed P/V_Ø_ of 0.12 kW m^−3^ of the shake flask. This was used to calculate the stirrer speed with Eq. 8 (*n* = 117 rpm). Accordingly, the stirring speed was increased from 100 to 250 rpm. After reaching 250 rpm, no further increase was performed to keep P/V_Ø_ in the STR in the range of P/V_Ø_ of the shake flasks. The Re-number is only slightly below 10^4^ (Re = 0.84 × 10^4^) for 250 rpm which is why a turbulent flow regime can be assumed. The OTR, VCD, and viability curves of the scale-up with constant P/V as a scale parameter are depicted in Fig. 4. The results from Fig. 1 are shown again for better comparability.

The OTR curves of all five cultivations in Fig. 4 are identical during the first increasing phase (ca. 3 days) until glutamine is depleted. Afterward, the second increasing phase starts. The decreasing phase starting at day 4 is approximately one day prolonged for the STR compared to the shaken devices. Therefore, the gradual OTR drop due to glucose depletion appeared on day 7 for the STR instead of day 6 as was seen for the shaken devices. Calculated OTR data between days 4.5 and 5.5 were falsified due to a technical problem with the off-gas cooler. Undefined excess water in the off-gas stream falsified the calculated OTR data and were therefore excluded from the graph. The DOT measurement is displayed in Fig. S5. The DOT drops to a minimum of about 50% which is absolutely tolerable as oxygen concentrations are usually maintained between 10 and 80% for CHO cell cultures [8].

The deviations between OTR progression of STR and shake flasks can be explained when keeping the difference between P/V_Ø_ and P/V_max_ for STRs in mind. As P/V_max_ was stated to be about 10 times higher than the P/V_Ø_, the results of the STR were compared to a shake flask cultivation with a ca. tenfold higher P/V (1.45 kW m^−3^) published previously [34] and shown in Fig. 4 (pink line and stars). Metabolic parameters for this cultivation are shown in Fig. S7. The OTR drop due to glucose depletion at day 7 of the STR is in between those of the reference cultivations in the shaken devices (day 6) and the cultivation with increased P/V_Ø_ (day 8). Thus, the results with the here-shown scale-up strategy are explainable and predictable regarding the OTR. The results can be confirmed by offline analyses, as shown in the following.

**Fig. 5:**
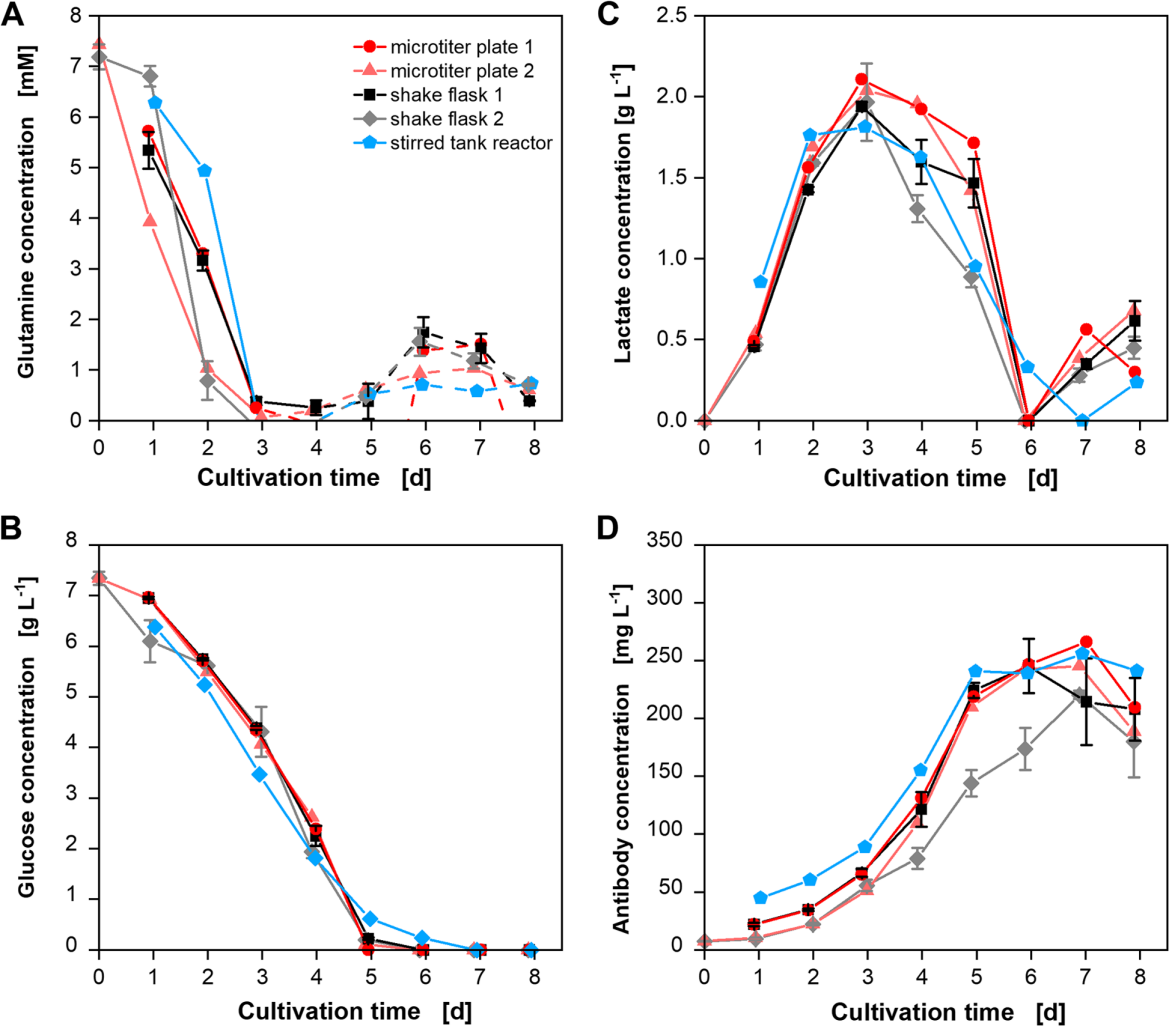
Offline measured metabolite and product concentrations of the cultivations shown in Fig. 4. CHO DP12 cell cultivations were performed in round 96-deep-well plates (dark and light red lines/circles and upward triangle), shake flasks (black and gray lines/squares and diamonds), and a stirred tank reactor (blue line and pentagon). Depicted are **A**) glutamine concentrations **B**) glucose concentrations **C**) lactate concentrations and **D**) IgG antibody titer

The VCD shown in Fig. 4B is comparable between the STR and the shaken devices. The viability of the STR is at a constant high level for the first three cultivation days before there is a slight decrease to around 80% for the next three days. These deviations could be due to the method of VCD and viability determination. They were determined by using the manual Neubauer chamber method for the STR cultivation while the automated CEDEX device was used for the other cultivations. As the manual method is known as imprecise, this could have led to inaccuracies. The time of the severe viability decrease is again the same for all cultivations. Next to OTR, VCD, and viability, different offline parameters were analyzed for all cultivations and are depicted in Fig. 5.

The glutamine concentrations in Fig. 5A demonstrate that the glutamine consumption was identical for all cultivations and is in accordance with the observations from Fig. 4 where the kink in the OTR curve appears for all of them at almost the same time (approximately after 3.5 days). After depletion, the measured values increase again (dotted lines) which is only due to uncertainties and inaccurate measurements for very low or no glutamine concentrations of the spectrophotometric kit used for determining glutamine concentrations. According to the manufacturer, the lower detection limit is at 0.543 mg L^−1^. Glucose (Fig. 5B) and lactate (Fig. 5C) consumption are in general also comparable between all cultivations in shaken devices and the STR even though the time of lactate depletion is slightly shifted backward for the STR. Lactate concentrations increase again for all cultures after depletion (cultivation days 6 / 7). At this time, the production of lactate in glutaminolysis and citric acid cycle is higher than its consumption. The most important aspect of a cultivation is the product production. Therefore, antibody concentrations were measured over the whole cultivation time. As can be seen from Fig. 5D, a titer of about 250 mg L^−1^ was reached in all five cultivations. This is the maximum known titer for this cell line stated by the supplier. All in all, the scale-up from the shaken devices to a STR by using a scale strategy concerning P/V could be shown to be successful.

To summarize, two different approaches of scale-up processes were performed within this study. The first one used OTR_max_ as scale-up parameter. This strategy focuses on sufficient oxygen supply within cultivation but does not concern hydrodynamics, shear forces or power input. The second scale-up strategy used P/V_Ø_ as scale-up parameter. With this strategy, hydrodynamics and different kinds of forces come into focus. Oxygen supply is not an issue in mammalian batch cultivations as the cells grow slowly and oxygen is usually available in excess. In contrast, previous studies in shake flasks and STRs showed that different levels of hydromechanical stress influence CHO cell growth and eventually also production. Therefore, a scaling strategy with constant P/V_Ø_ is more sensible for mammalian cells and improves comparability to shaken devices. However, in perfusion processes with high cell densities oxygen availability must be considered.

## Conclusion

This study addressed the question of whether a scale-up from small shaken devices to a STR is possible for CHO cells. The scale transfer between round 96-deep-well MTPs with 1 mL filling volume and shake flasks could be performed using CHO DP12 cells with OTR_max_ as the scale-up parameter. The OTR curves of the corresponding experiments were nearly identical. Furthermore, it was shown that the filling volume of the round 96-deep-well MTPs could be reduced by 60% to 400 µL, leading to the same results as in shake flask cultivations. Moreover, it was demonstrated that the CHO DP12 cells can be cultivated in square 96-deep-well plates as well. The results for filling volumes between 400 µL to 900 µL are comparable to the results obtained in the round well-plates and the shake flasks. Due to the higher power input and baffling effects, they could be an even better choice for scale-up approaches to STRs than round well plates. However, further investigations must be carried out. The transfer of cultivation to a STR by using an OTR_max_ based scale-up strategy was not successful. The resulting hydromechanical stress was too high in the STR. Therefore, a constant P/V_Ø_ as scale-up parameter while ensuring a sufficient oxygen supply (DOT > 50%) was shown to be suitable for a scale-up to a STR. A detailed analysis of P/V_Ø_ in shake flasks was consulted to perform a data-driven scale-up. The cultivation results in the form of the OTR were predictable when considering that an average and maximal local P/V are present in the STR but the difference is negligible in shaken devices under laminar conditions. Common offline parameters like VCD, viability, glucose-, lactate, and glutamine concentrations were very similar in all three cultivation devices. Strikingly, also the antibody titer was the same for all cultivations when considering that the volumes were varied by three orders of magnitude across the scales.

This study showed that a data-driven scale-up from 96-deep-well-MTPs with a filling volume of a minimum of 400 µL to a STR with 600 mL working volume was successful, meaning that the same cultivation conditions and the same final antibody titer were achieved. This may lead to future experiments with CHO cells being carried out on a small shaken scale and subsequently being predictably reproduced in a STR. The method can also be adapted for other, possibly more sensitive animal cells in the future. It is moreover conceivable that the shaken systems are used as scale-down models for larger systems e.g. simulate heterogeneity effects at large-scale.

## Data Availability

All data is available on request to the corresponding author.
